# Blood ATX(N) Biomarkers and Cognitive Dysfunction in Severe Mental Illnesses

**DOI:** 10.3390/ijms27104260

**Published:** 2026-05-11

**Authors:** Daniela Crescenti, Irene Petracci, Andrea Cesareni, Giuliano Binetti, Barbara Borroni, Roberta Ghidoni

**Affiliations:** 1 Molecular Markers Laboratory, IRCCS Istituto Centro San Giovanni di Dio Fatebenefratelli, 25125 Brescia, Italy; dcrescenti@fatebenefratelli.eu (D.C.); ipetracci@fatebenefratelli.eu (I.P.); bborroni@fatebenefratelli.eu (B.B.); 2 Psychiatry Unit, IRCCS Istituto Centro San Giovanni di Dio Fatebenefratelli, 25125 Brescia, Italy; acesareni@fatebenefratelli.eu; 3 MAC-Memory Clinic and Molecular Markers Laboratory, IRCCS Istituto Centro San Giovanni di Dio Fatebenefratelli, 25125 Brescia, Italy; gbinetti@fatebenefratelli.eu; 4 Department of Clinical and Experimental Sciences, University of Brescia, 25123 Brescia, Italy

**Keywords:** amyloid beta, ATX(N) system, blood biomarkers, cognitive impairment, differential diagnosis, glial fibrillary acidic protein, neurodegenerative dementias, neurofilament light, severe mental illnesses, tau

## Abstract

Psychiatric disorders, such as major depressive disorder (MDD), bipolar disorder (BD), and schizophrenia (SZ), comprise a heterogenous group of severe mental illnesses (SMIs) characterized by disturbances in cognition, emotional regulation, or behavior. Cognitive impairment represents an accompanying feature of many SMIs, often interfering with or limiting essential daily life activities. SMIs arise from a complex interplay of genetic, epigenetic, developmental, and environmental factors that disrupt neural and cellular processes. SMIs often present with overlapping symptoms and sometimes co-occur, making misdiagnosis a common clinical challenge. To date, there is a lack of reliable and specific biological markers to aid in the differential diagnosis of cognitive impairment in SMIs and for distinguishing neurodegenerative dementias from SMIs with overlapping symptoms. In this context, blood-based biomarkers of the ATX(N) system associated with cognitive deficits in neurodegenerative diseases, such as neurofilament light chain (NfL), glial fibrillary acidic protein (GFAP), amyloid beta (Aβ), and tau proteins, may help to understand the biological basis of cognitive dysfunction in SMIs and support differential diagnosis. This narrative review summarizes the current evidence on the application of blood-based biomarkers of neurodegenerative dementias in SMIs and their association with the cognitive deficits observed in these conditions, as well as their relevance for differential diagnosis, disease monitoring, and the evaluation of treatment efficacy in psychiatric disorders.

## 1. Introduction

Cognitive functioning, which refers to the mental processes necessary to acquire knowledge, think, reason, understand, and remember, is essential for day-to-day activities, to interact with the world and solve problems [1]. When compromised, it can severely impact an individual’s quality of life because daily tasks become extremely challenging and debilitating. Cognitive impairment is a feature of many neuropsychiatric disorders, which substantially contribute to the psychological, economic, and medical burden on families and society [2].

Severe mental illnesses (SMIs) are defined as syndromes characterized by disturbances in an individual’s cognition, emotional regulation, or behavior, each of which is present to a high but variable degree of clinical severity, as outlined in the *Diagnostic and Statistical Manual of Mental Disorders*, 5th edition, text revision (DSM-5-TR) [3]. Major depressive disorder (MDD), bipolar disorder (BD), and schizophrenia (SZ) are among the most commonly diagnosed and debilitating SMIs. They represent leading causes of disease-related burden, especially due to their recurrent course and life-long duration. MDD affects approximately 4% of the global population (about 332 million individuals), including 5.7% of adults and 5.9% of those aged 70 years and older, and is approximately 1.5 times more prevalent in women than in men [4,5]. MDD’s prevalence, particularly among adolescents and young adults, has risen significantly in recent decades, further worsened by the COVID-19 pandemic [6,7]. An estimated 37 million people (0.5% of the global population), including approximately 34 million adults, live with BD worldwide [8,9]. BD can occur across the lifespan but typically shows a bimodal age distribution, with peaks in late adolescence/early adulthood (15–24 years) and mid-adulthood (45–54 years) [10]. SZ, which usually emerges in late adolescence or early adulthood and tends to occur earlier among men than women, affects approximately 23 million people worldwide (0.3% of the global population) [11,12]. Although less prevalent than other mental disorders, SZ is a severe, lifelong condition that incurs significant economic costs and requires long-term treatment [11].

Primary SMI-related cognitive dysfunction is typically characterized by an early onset, often emerging within the first years or decades of illness, and a relatively stable or slowly progressive longitudinal course, with fluctuations largely reflecting clinical stability or treatment effects [13,14]. Cognitive performance may also show partial improvement with optimal symptom control and medication adjustment. However, the deficits can worsen with age or repeated hospitalizations and prolonged medication use, such as antipsychotics [15]. SMIs are associated not only with early cognitive deficits and a faster rate of cognitive decline in later life, often leading to premature loss of independence [16,17], but also with an increased risk of accelerated cognitive decline with aging, resulting in a higher risk of later-life dementia than the general population [18,19,20]. In a large population study, Richmond-Rakerd et al. observed that, among patients with dementia, those with a mental disorder developed dementia on average 5.60 years (95% CI 5.31–5.90) earlier than those without a history of mental illness [21]. The decline arising from an underlying psychiatric illness must be carefully differentiated from a true add-on neurodegenerative disorder, such as Alzheimer’s disease (AD) or other forms of dementia, especially in older adults, where cognitive symptoms could be either a consequence of the mental illness itself or the prodromal manifestation of dementia [22]. The presence of a co-occurring neurodegenerative disorder in an individual with SMI alters the cognitive and functional trajectory, resulting in faster, progressive, and more persistent cognitive decline [23,24]. The need of proper differential diagnosis between cognitive impairment secondary to SMIs or neurodegenerative conditions has prompted the development of biomarkers that could enable earlier detection and intervention, potentially improving or even preventing disease progression.

Peripheral blood is one of the most widely explored matrices for biomarker discovery due to its non-invasive collection, minimal risk to patients, potential for early detection, cost-effectiveness, and variety of biomolecules that reflect changes in distant tissues and organs.

This narrative review offers an overview of the current evidence on the role of blood biomarkers commonly used for neurodegenerative dementias in SMIs, focusing on their association with cognitive deficits, as well as their relevance for differential diagnosis, disease monitoring, and the evaluation of treatment efficacy in psychiatric disorders.

## 2. Molecular and Clinical Features of SMIs

SMIs arise from a complex interplay of genetic, epigenetic, developmental, and environmental factors that collectively disrupt neural and cellular systems [25,26]. These factors affect multiple cognitive domains and contribute to the behavioral and cognitive symptoms typical of these disorders. At the molecular level, key mechanisms include dysregulation of neurotransmitter systems and impaired synaptic plasticity, alongside neuroinflammation, which further contributes to neural damage and cognitive decline [27,28]. Other contributing factors include vascular pathology, mitochondrial dysfunction, dysregulation of the hypothalamic–pituitary–adrenal (HPA) axis, and disturbances in the gut–brain axis [29,30,31,32]. SMIs are highly heritable and polygenic in nature, with hundreds of genetic variants contributing to disease risk, while environmental influences further shape vulnerability through epigenetic mechanisms [33].

Despite MDD, BD, and SZ sharing overlapping symptoms such as psychosis, mood changes, and cognitive issues, they differ in their core psychopathological domains, illness course, and clinical trajectories. MDD and BD traditionally belong to the mood spectrum disorders. They are primarily characterized by persistent and intense emotional fluctuations, ranging from extreme highs (mania/hypomania) to lows (depression), and generally present an episodic course with variable inter-episode recovery [34,35,36]. In particular, MDD is characterized by short- and/or long-term depressed mood and anhedonia, accompanied by symptoms such as feelings of guilt, low energy, poor concentration, psychomotor retardation or agitation, sleep and appetite disturbances, and suicidal thoughts [37]. These symptoms can significantly alter how individuals perceive and interact with their environment, often leading to difficulties in social functioning as well as in cognitive and intellectual performance. Several hypotheses have been proposed to explain the multifactorial etiology of MDD. These include: dysregulation of the HPA axis; alterations in monoaminergic neurotransmission; and dysregulation of inflammatory processes and mediators. Genetic susceptibility also contributes to disease risk, often interacting with environmental influences. In addition, structural and functional brain changes (reduced densities of glial cells in key brain regions, such as the prefrontal cortex, hippocampus, and amygdala) have been observed, alongside psychosocial factors such as exposure to traumatic or chronic stressors [38]. On the other hand, BD is characterized by marked fluctuations in mood, energy, and daily functioning, with alternating episodes of mania or hypomania (elevated mood, increased energy, impulsivity, and reduced need for sleep) and depression (low mood, fatigue, anhedonia, and feelings of worthlessness). Onset typically occurs in late adolescence or early adulthood and the course is often progressive, with increasing severity and recurrence of episodes over time [39]. Its pathophysiology involves dysregulation of neurotransmitter systems, particularly glutamatergic and noradrenergic pathways, driven in part by genetic variants that affect synaptic signaling, neuronal development, plasticity, and neuroinflammatory processes. Environmental factors, including stress, trauma, circadian rhythm disturbances, and metabolic comorbidities such as obesity, cardiovascular disease, and diabetes, can interact with genetic vulnerability and contribute to the triggering of mood episodes [40]. Such interactions are also considered bidirectional, suggesting the existence of interconnected pathogenic networks between SMIs and somatic diseases, including those associated with a high risk of mortality [41].

Differently from MDD and BD, SZ is primarily classified within the psychotic spectrum disorders. It typically follows a chronic or progressively deteriorating course, leading to substantial and sustained functional impairment. Clinically, SZ affects multiple domains, including thinking, perception, emotion, language, self-experience, and behavior, with core symptoms such as hallucinations (often auditory), delusions, disorganized speech and behavior, and cognitive deficits [42]. Although the neurobiological mechanisms are not fully understood, SZ has a strong genetic component involving common and rare variants [43] thought to disrupt key biological pathways, including protein glycosylation and neurotransmitter signaling [44,45]. Among these, dopaminergic dysfunction is central and associated with positive (e.g., delusions and hallucinations) and negative symptoms (e.g., cognitive dysfunction, lack of motivation) [46].

## 3. Blood-Based Biomarkers in SMIs

MDD, BD, and SZ often exhibit cognitive deficits that resemble early manifestations of neurodegenerative processes, complicating diagnosis and management. This phenotypic overlap underscores the urgent need for biomarkers that can accurately define the biological mechanisms underlying cognitive decline. Such biomarkers are essential for early diagnosis and patient stratification; for refining differential diagnosis and clinical monitoring in patients presenting with cognitive symptoms, allowing for timely differentiation between SMIs and underlying neurodegenerative processes; for assessing disease progression, treatment efficacy and guiding targeted interventions, especially in late-onset forms where cognitive decline may be an early sign of a neurodegenerative pathology.

In this context, the AT(N) system may offer a biological approach to understand cognitive dysfunction in SMIs. The AT(N) research framework divides the major biomarkers used in AD research into three categories according to the underlying pathophysiological processes they measure (i.e., amyloidopathy (A), tauopathy (T), and neurodegeneration and neuronal injury (N)) [47]. The expanded ATX(N) framework broadened this perspective by incorporating additional, non-specific biological processes, such as astrocytic activation and inflammation (I), which contribute to neurodegenerative pathology [48,49]. The ATX(N) system captures fundamental biological processes that may also be implicated in mood and psychotic disorders, namely altered proteostasis, cytoskeletal and synaptic dysfunction, neuronal and glial damage, although with distinct temporal dynamics and quantitative expression. The ATX(N) framework may provide a cross-cutting conceptual and biological model to study cognitive decline and neuroprogression in SMIs, to identify individuals with accelerated brain aging, to differentiate SMI-related cognitive impairment from prodromal dementia, and to inform precision medicine strategies aimed at preventing or slowing cognitive decline.

### 3.1. ATX(N) Biomarkers in SMIs

Neurofilament light chain (NfL), glial fibrillary acidic protein (GFAP), amyloid beta (Aβ), and tau proteins are well-established ATX(N) biomarkers of neurodegenerative dementias [50]. Blood-based measurements of these biomarkers have recently been formally incorporated into the revised criteria of the National Institute on Aging and Alzheimer’s Association (NIA-AA) workgroup for the biological definition, diagnosis, and staging of AD [49]. Emerging evidence have also associated these biomarkers to the cognitive deficits observed in SMIs, suggesting potential shared neurobiological mechanisms underlying cognitive dysfunction across both neurodegenerative and psychiatric conditions.

NfL is the most abundant and soluble component of neuronal axons. Neurofilaments are released into the cerebrospinal fluid (CSF) and blood as a result of processes causing axonal injury. This makes NfL levels a reliable biomarker of neuronal brain injury and degeneration [51]. NfL is a sensitive but not-specific marker of neuroaxonal damage in different neurological conditions, including neurodegenerative disorders, such as amyotrophic lateral sclerosis (ALS), AD, frontotemporal dementia (FTD), multiple sclerosis (MS), traumatic brain injury (TBI), and also SMIs [52,53,54,55,56,57]. At postsynaptic sites, neurofilaments differentially regulate glutamatergic and dopaminergic neurotransmission, potentially influencing synaptic function and cognition in neuropsychiatric diseases [58,59,60].

GFAP is a major component of the mature astrocyte cytoskeleton, and it is considered a marker of astrocyte activation and reactive gliosis [61]. In neurodegenerative diseases such as dementia, elevated blood GFAP levels are robustly associated with cognitive decline and disease progression, reflecting underlying neuroinflammatory and neurodegenerative processes [62,63,64]. Evidence suggests that astrocytic dysfunction and neuroinflammation may also play a role in psychiatric disorders [65,66], although the relationship between GFAP and cognitive impairments in these patients remains uncertain, as most existing data are indirect or derived from postmortem brain studies [67,68,69,70]. Decreased GFAP mRNA and protein expression have been observed in the anterior cingulate cortex in BD and SZ [67], in the prefrontal cortex in SZ [71], and in the ventral prefrontal cortex [72], cerebellum [70], thalamus and caudate nucleus [73], and locus coeruleus of individuals with MDD [74].

Aβ is a self-aggregating peptide typically associated with the pathogenesis of AD and related amyloidopathies. Amyloid accumulation may also be associated with the onset and course of SMIs, potentially through neurobiological processes such as neuroinflammation, neurodegeneration, vascular injury, blood–brain barrier dysfunction, synaptic plasticity, and functional connectivity, which in turn may affect neural networks associated with SMIs [75,76,77]. Indeed, elevated brain amyloid deposition has been associated with an increased risk of neuropsychiatric symptoms in subjects with mild cognitive impairment (MCI), suggesting that amyloidosis may drive both cognitive and psychiatric symptoms [78]. Moreover, altered amyloid metabolism connected to neurodevelopmental processes and neuronal degeneration is common in SMIs [79,80,81,82,83,84]. Thus, Aβ levels may represent a potential biomarker of altered neurodevelopment and accelerated neurodegeneration in SMIs.

Tau, a microtubule-associated protein, is a central biomarker in neurodegenerative diseases, especially AD, where its hyperphosphorylation and aggregation are hallmarks of pathology. Elevated CSF and plasma tau, especially the phosphorylated isoforms (e.g., p-tau181 and p-tau217), are robust biomarkers for AD, correlating with amyloid pathology and cognitive decline [85,86]. Tau dysfunction extends well beyond neurofibrillary tangle formation, and tau-related biomarkers may reflect early cytoskeletal instability, neuronal distress, and synaptic dysfunction [87]. While tau’s role in neurodegeneration is well established, its relevance in SMIs is less clear.

#### 3.1.1. Major Depressive Disorder

Cognitive deficits are extremely common in MDD patients [88]. The impairments mainly address executive function, learning and memory, processing speed, attention and concentration, and are shown to persist even after remission of mood symptoms [89].

Although individuals with SMIs generally do not exhibit the marked elevation in blood biomarkers of neuroaxonal injury and astroglial activation typical of neurodegenerative diseases, NfL and GFAP concentrations appear to be associated with these impairments in MDD, albeit with varying levels of consistency across studies. In particular, increased NfL levels in the blood have been associated with poorer cognitive performance, especially in working memory and executive function, in MDD patients [90,91,92,93,94]. NfL concentrations have been linked to global cognitive decline [95], and may mediate the relationship between depressive symptoms and cognitive decline in older adults [92]. Indeed, higher NfL levels correlated with greater depressive symptoms [90,96] and reduced cognitive processing speed, as measured by the Digit Symbol Substitution Task (DSST) score [90]. Additionally, NfL concentrations have been shown to predict DSST performance in individuals with MDD [90], and have been linked to an increased risk of lifetime suicidal behaviors with every 10 pg/mL increase in the NfL levels [97]. However, despite these associations, findings regarding group differences between MDD patients and healthy controls (HC) appear heterogeneous. Several studies showed elevated blood NfL levels in MDD and treatment-resistant depression patients compared to HC [90,94,98,99,100], especially in those with cognitive deficits or with an underlying neurodegenerative pathology [91], whereas other studies, including a large-scale meta-analysis, reported no significant differences in NfL levels between MDD patients and HC [53,101,102,103,104,105]. This variability may reflect differences in sample characteristics, illness stage, or pathophysiological mechanisms, and the presence of comorbid or underlying neurodegenerative processes.

Similarly, GFAP has been linked to both cognitive dysfunction [106,107,108] and depressive symptoms severity in MDD [109], further supporting the role of astroglial pathology in the disorder [108]. In particular, elevated plasma/serum GFAP levels have been associated with poorer cognitive performance on the Montreal Cognitive Assessment (MoCA) and DSST, especially in older adults [106,107]. Notably, individuals with late-onset MDD showed an increased density of GFAP-positive astrocytes in the dorsolateral prefrontal cortex compared to age-matched controls and young-onset MDD [110,111]. Nevertheless, while some data suggest a stepwise increase in GFAP and NfL levels alongside MDD severity [100], findings on group differences in circulating GFAP are conflicting—as previously seen with NfL [100,103,105,109,112,113]. Indeed, some studies report no differences or even lower GFAP levels in MDD populations [103,105], suggesting that the extent of astroglial dysfunction may vary across clinical subgroups.

Beyond neuroaxonal and astroglial markers, accumulation of amyloid and tau proteins has been observed in the brain of MDD patients [114,115,116,117,118]. Specifically, depression severity has been positively correlated with tau accumulation in the inferior temporal lobe and entorhinal area [119], while amyloid deposition has been observed in the precuneus and parietal lobe [120]. These regions are also critical areas affected by tau and Aβ pathology in AD [121]. Furthermore, altered circulating levels of Aβ have also been associated with MDD [114,122,123]. In particular, several studies reported either reduced Aβ42/Aβ40 ratios [114,122], increased Aβ40 and decreased Aβ42 levels [114,124], or increased Aβ40/Aβ42 ratios [125] in the serum or plasma of late-life MDD compared to HC, even in younger patients [114,124]. These alterations in Aβ profile and metabolism have been associated with more severe depressive symptoms [106,122,126,127]; pronounced cognitive deficits in visuospatial ability, executive function, and particularly memory [106,128]; and a higher long-term risk of AD onset [122]. Alterations in biomarkers of tau pathology have also been associated with cognitive impairment [106] and higher depressive symptoms, particularly in individuals with concurrent MCI [96]. However, not all evidence is consistent as other large-scale and meta-analytic evidence have failed to confirm the association between late-life depressive symptoms and plasma biomarkers of AD pathology, axonal injury, or astrocytic activation [129], highlighting existing heterogeneity in the literature.

Taken together, cognitive impairment in MDD might be associated with multiple, potentially overlapping biological processes, including neuroaxonal injury, astroglial activation, and, in some cases, AD-related pathology [106]. A key theme emerging is that these associations are more pronounced in specific subgroups, particularly older adults, individuals with more severe or treatment-resistant depression, and those with comorbid cognitive impairment or MCI.

#### 3.1.2. Bipolar Disorder

Cognitive impairment in BD is a well-established but highly heterogeneous feature, with deficits particularly in verbal learning, attention, and executive function [130,131]. Importantly, cognitive profiles in BD vary widely across individuals, ranging from global and multi-domain impairments to more selective deficits, while a substantial proportion of patients (32–48%) remain relatively cognitively preserved [132,133]. This variability represents a key feature of BD and might reflect differences in illness course, neurobiological mechanisms, and clinical subtypes.

Across studies, elevated blood NfL levels have been consistently associated with worse cognitive performance in BD, with impairments in global cognition, executive function, working memory, and processing speed [93,134,135], and have also been linked to white matter microstructure integrity, suggesting a role in neuronal connectivity remodeling processes [136]. Moreover, NfL concentrations tend to increase with illness duration, supporting the hypothesis of progressive neuroaxonal damage over time [137], and to be higher in BD patients who experience an affective episode [138]. However, while several studies and meta-analytic evidence report mildly but significantly higher NfL levels in BD compared to HC [104,105,136,137,139], even in children and adolescents [140], other studies have failed to replicate these findings [101,109,138].

In contrast, evidence regarding GFAP remains more limited and inconsistent, suggesting that astroglial involvement may characterize specific subgroups of BD patients within the disorder’s heterogeneity. In fact, while some studies reported elevated GFAP levels in BD [112,137], particularly in BD patients with later onset [137], supporting a role for astrocytic dysfunction and neuroinflammatory processes, others found no significant differences compared to HC [109].

The role of AD-related biomarkers in BD, particularly Aβ and tau, is less clearly defined. Studies in young BD populations reported no significant differences in plasma Aβ40, Aβ42, Aβ42/Aβ40 ratio or tau levels compared to HC [138], nor consistent associations with different cognitive domains, including global cognition, verbal memory, executive function, psychomotor speed, and sustained attention [141]. However, emerging evidence suggests that specific subgroups, such as young BD patients with accelerated epigenetic aging, may exhibit altered amyloid profiles, specifically reduced plasma Aβ42/Aβ40 ratios [142]. Furthermore, while tau-related changes have not been consistently linked to cognitive deficits in vivo, neurotoxic p-tau231 conformers have been observed in postmortem brains of BD patients [143]. Although psychiatric disorders are not classically associated with tauopathy, some evidence raised the possibility of underlying tau-related neurodevelopmental or neurodegenerative processes in early-onset psychosis and BD [143,144]. However, causal relationships cannot be inferred and warrant further investigation.

Taken together, the available evidence indicates an association between cognitive impairment and biomarkers of neuroaxonal injury (i.e., NfL), with more limited and heterogeneous support for astroglial and AD-related processes (GFAP and Aβ and tau, respectively). Moreover, these findings suggest that biomarker alterations, and their relationship with cognition, may be more pronounced in specific BD subgroups, particularly in those with a more severe or progressive disease course, rather than representing a uniform feature of the disorder. However, a key overarching theme is the substantial heterogeneity across studies, which likely reflects differences in age, illness duration, clinical status, and biological vulnerability.

Relevant evidence on Aβ in BD is reported in CSF studies, but their discussion lies beyond the scope of the present review [79,138,141,145].

#### 3.1.3. Schizophrenia

Cognitive impairment is also a central feature of SZ. It affects approximately 80% of individuals with SZ and typically manifests as deficits in processing speed, memory, attention, and executive functions such as planning and decision-making [45].

In SZ, the evidence supporting an association between NfL levels and cognitive performance remains limited and somewhat inconsistent. Some studies suggest that NfL may reflect aspects of clinical severity rather than cognition per se. For instance, Popovic et al. reported increased NfL levels in acutely ill, unmedicated SZ patients compared to HC, with NfL levels positively correlating with symptom severity, as measured by the Positive and Negative Syndrome Scale (PANSS), and inversely correlating with global functioning assessed by the Global Assessment of Functioning (GAF) scale [146]. Similarly, Nisha et al. found a negative association between serum NfL levels and negative symptom severity in individuals at clinical high-risk for SZ. However, they did not observe any significant relationship between NfL concentrations and cognitive performance assessed using the Repeatable Battery for the Assessment of Neuropsychological Status (RBANS) [147]. Other findings point to potential differences according to treatment response. In particular, patients treated with clozapine have been reported to exhibit higher NfL levels, a worse clinical outcome, and greater cognitive impairment compared to SZ patients responding to first-line antipsychotics [66]. Nevertheless, the overall literature does not consistently demonstrate elevated NfL levels in SZ. Several studies did not find significant differences between SZ patients (including those treated with clozapine) and HC [90,101,109,148], or among individuals in the early and prodromal stages of psychosis, including untreated first-episode psychosis and high-risk subjects [147]. Conversely, other investigations reported higher plasma NfL levels in SZ and clozapine-treated SZ [66], and in the serum of patients with first-episode psychosis compared to HC [149]. Elevated serum NfL levels have also been described in children and adolescents with SZ compared to age-matched HC [140]. Even in studies where mean NfL levels did not significantly differ from reference controls, a greater proportion of SZ patients exhibited elevated NfL values, and NfL concentrations were positively correlated with illness duration [90]. Taken together, these findings suggest that while NfL levels may be related to clinical severity, illness progression, or treatment resistance in SZ, its association with cognitive impairment remains unclear and warrants further investigation.

Astroglial alterations have also been investigated in SZ. Reactive astrogliosis was found in approximately 70% of patients with SZ, with GFAP levels varying among patients with the disease [69]. Yet, research investigating peripheral GFAP levels in SZ remains limited and has yielded inconsistent findings. Increased plasma levels of GFAP have been observed in SZ patients, as well as in a subgroup of chronic patients treated with clozapine [66], and in ultra-treatment-resistant SZ patients compared to HC [150]. In contrast, other investigations suggest a different pattern. Lower serum GFAP levels were found in SZ compared to HC, with no significant differences between first-episode and relapsed patients or changes after treatment [151], findings consistent with previous reports [152]. Overall, these findings point to a complex and not yet fully understood pattern of astroglial and neuroaxonal biomarker alterations in SZ, with substantial variability depending on illness stage, treatment resistance, and diagnostic subgroup. Additionally, the literature is notably lacking in studies examining the association between peripheral GFAP concentrations and cognitive deficits in SZ.

Tau-related mechanisms have also been explored. Lower circulating tau and p-tau levels have been reported in the serum of adults with SZ [153]. Consistent with this, reduced total tau levels were also observed in the plasma of adolescents with early-onset psychosis, where tau concentrations correlated with orbitofrontal cortex surface area and were not associated with antipsychotic use or dosage [144]. A recent randomized controlled trial involving 40 individuals with SZ treated with transcranial Direct Current Stimulation (tDCS) demonstrated a significant increase in serum p-tau levels in the active tDCS group compared to the sham group, alongside a significantly greater improvement in working memory performance (i.e., Digit Span test) [154]. These findings suggested that neuromodulation with tDCS may enhance memory function in SZ patients by modulating tau levels, and that serum p-tau may represent a candidate peripheral biomarker of treatment response [154]. Tau hyperphosphorylation is usually considered pathological in neurodegenerative disorders. This discrepancy suggests that the relationship between tau and cognitive function in SZ may follow a different pattern or be mediated by mechanisms distinct from those implicated, for example, in AD. However, additional research is necessary to establish the role of tau protein in SZ and its impact on cognitive impairments. To the best of our knowledge, although Aβ levels have been assessed in CSF and postmortem brain tissue [79,82,83,155,156], we could not find any studies examining Aβ levels in the blood of SZ patients.

A summary of the evidence on the associations between blood ATX(N) biomarkers and cognition, alongside associations with disease severity and illness duration, in patients with MDD, BD, and SZ is presented in Table 1.

#### 3.1.4. Differential Diagnosis Between SMIs and Neurodegenerative Dementias

Neurodegenerative dementias and SMIs, more frequently in late-onset forms, often present with overlapping symptoms, including mood disturbances and cognitive impairment, and sometimes co-occur, making misdiagnosis a common clinical challenge. Misdiagnosis can delay proper care, lead to inappropriate medications and worsen outcomes. For example, FTD, particularly the behavioral variant (bvFTD), can closely resemble various psychiatric conditions due to its prominent behavioral features. About 50% of bvFTD patients initially receive a psychiatric diagnosis, with an average diagnostic delay of 5–6 years from symptom onset. Similarly, patients with SMIs are frequently misdiagnosed with bvFTD [157]. Dementia with Lewy bodies (DLB) can be mistaken for SZ [158] due to its core clinical features, which include visual hallucinations, depression, apathy, and delusions, typically occurring within the first 5 years since diagnosis [159]. Considering the overlapping of symptoms and presentations, differential diagnosis between neurodegenerative dementias and SMIs is fundamental to provide appropriate medical care, delay disease progression, or manage the condition effectively.

Blood NfL, in particular, and GFAP have shown promise in differentiating psychiatric disorders from neurodegenerative diseases. Indeed, NfL levels were found to be significantly elevated in neurodegenerative or neurological disorders compared to SMIs, supporting its potential utility as a diagnostic biomarker for the often-challenging differentiation of these conditions [53,95,157,160,161]. In particular, a growing body of evidence demonstrates the potential use of NfL for the differential diagnosis between SMIs and FTD, in particular bvFTD [101,104,112,157,160,162,163,164]. Collectively, blood NfL has demonstrated an appropriate level of sensitivity and specificity in differentiating bvFTD from psychiatric disorders, with areas under the curve (AUCs) ranging from 0.79 to 0.98 (65–100% sensitivities, 69–96% specificities), although with differences in diagnostic performance across studies [165]. A study by Eratne et al. comparing plasma GFAP in BD, MDD, SZ, HC, and bvFTD found elevated GFAP levels in bvFTD compared to all SMIs and HC [112], as also described by Katisko and colleagues [166]. Katisko found that elevated serum GFAP in bvFTD enabled differentiation of patients with bvFTD and primary psychiatric disorders (PPD) with an AUC equal to 0.815 [95% CI 0.733–0.896], 80.3% sensitivity and 69.0% specificity, and that combining NfL with GFAP increased the diagnostic performance in distinguishing all frontotemporal lobar degeneration (FTLD) patients from PPD (AUC 0.890 [95% CI 0.823–0.956], 83.3% sensitivity, 87.9% specificity) [166]. Eratne et al. and de Boer et al. found a similar diagnostic utility of plasma/serum GFAP in discriminating bvFTD from PPD (AUC 0.86 [95% CI 0.76–0.95] and AUC 0.787 [95% CI 0.737–0.837], respectively), but did not find any benefit in combining NfL with GFAP (AUC_NfL_ 0.95 and 0.872 vs. AUC_NfL+GFAP_ 0.90 and 0.878, respectively), supporting the use of NfL as a standalone biomarker in the distinction between young-onset dementias, especially bvFTD, and PPD [112,163,167].

## 4. Materials and Methods

### Literature Search Strategy

This narrative review was conducted with the aim of providing an overview of the available and most updated literature on ATX(N) blood biomarkers and cognitive dysfunction in SMIs. A non-systematic targeted literature search was performed across major electronic databases, including PubMed, Scopus, and Web of Science, consulted up to February 2026. The search focused primarily on original research articles, meta-analyses, as well as relevant systematic review articles, published in English within the last 15 years. Studies were included based on relevance to the objectives of the review. Older seminal references were included when historically significant or particularly influential. Selected review articles were also incorporated to provide background and contextual support. The reference lists of relevant articles were additionally screened to identify further studies for inclusion. Articles that were not peer-reviewed, published as conference abstracts, or with unavailable full-text were excluded.

## 5. Conclusions

Psychiatric diagnosis is still entirely clinical, based on structured interviews, observed behavior, and diagnostic criteria [168]. Currently, there is a lack of reliable and specific biological markers to aid in the differential diagnosis of cognitive impairment in SMIs. As a source of biomarkers, blood is one of the most promising.

Across SMIs, blood-based ATX(N) biomarkers may offer a coherent framework for delineating the biological substrates of cognitive dysfunction and enhancing diagnostic accuracy in clinically complex presentations. Converging evidence across MDD, BD, and SZ indicates that circulating markers of axonal injury (NfL) and astroglial activation (GFAP) show the most reproducible associations with cognitive impairment, supporting the hypothesis that neuroaxonal vulnerability and glial dysregulation contribute to neuroprogressive trajectories in subsets of individuals with SMIs. In contrast, alterations in amyloid- and tau-related pathways appear less consistent and more phenotype-specific, emerging predominantly in older adults, treatment-resistant cases, or individuals with comorbid cognitive impairment. Overall, the most consistent evidence emerges in MDD, whereas findings in BD and, in particular, in SZ remain limited or inconclusive. In MDD, converging data indicate that increased blood NfL levels, and to a lesser extent GFAP levels, are associated with cognitive impairments and greater symptom severity. In BD, the most consistent evidence concerns NfL, with higher circulating levels associated with poorer cognitive performance, longer illness duration, and acute illness phases, supporting the involvement of progressive neuroaxonal injury in a biologically vulnerable subset of patients. However, current study designs do not allow firm conclusions regarding whether these biomarker alterations reflect state- or trait-dependent processes across the full course of illness. Most available evidence derives from cross-sectional cohorts or studies lacking systematic longitudinal assessment across distinct clinical phases, limiting inferences about temporal dynamics and within-individual trajectories. Nevertheless, these observations may provide a biological rationale for future investigations.

ATX(N) biomarkers also hold substantial promise for addressing one of the most persistent clinical challenges: the differential diagnosis between SMIs and neurodegenerative dementias. Symptom overlap—particularly in late-onset presentations—frequently leads to misdiagnosis, as shown by the slightly high proportion of patients with bvFTD or DLB who initially receive psychiatric labels, and vice versa. In this context, blood NfL has demonstrated strong standalone diagnostic performance, with AUCs often exceeding 0.80–0.95 in distinguishing bvFTD from PPD.

Establishing the clinical utility, predictive value, and cost-effectiveness of ATX(N) biomarkers across psychiatric, primary care, and memory clinic settings will be essential to determine their translational relevance and support their progression from exploratory findings to clinically actionable tools.

## Figures and Tables

**Table 1:** Summary of studies examining the associations between blood ATX(N) biomarkers and cognition, disease severity, and duration in MDD, BD, and SZ patients.

	Cognitive Domains Affected	Blood Biomarker	Association with Cognitive Impairment	Biomarker Association with Cognition	Biomarker Association with Disease Severity and Duration
Major depressive disorder (MDD)	Executive function, learning, memory, processing speed, attention, concentration	NfL	Yes	Negative correlation with cognitive processing speed (DSST score) [90] Negative correlation with global cognition (MMSE and comprehension domain) in MDD clustered with neurodegenerative disorders [91] Associations with semantic memory, executive function, processing speed (AFT and DSST score) [92] Positive association with deficits in executive function [94]	Positive correlation with severity of depressive symptoms (HAMD score) [90] Positive association with ↑ depressive symptoms (GDS score) [96] No significant association with late-life depressive symptoms (GDS-15 score) [129]
		GFAP	Yes	Negative association with global cognition and executive function (MoCA and DSST scores) [106,107]	Positive correlation with severity of depressive episodes (MADRS score) [109] No significant association with late-life depressive symptoms (GDS-15 score) [129]
		Aβ	Yes	Negative correlation with MMSE score [117,118] ↓ Aβ42 levels associated with ↓ ability in facial memory test [106] ↑ Aβ40/Aβ42 ratio associated with ↓ memory (Delayed Recall score), visuospatial ability (Block Design), and executive function (Trails B score) [128]	↑ depressive symptoms (HADS-D score) associated with ↑ Aβ40 levels and ↓ Aβ42/Aβ40 ratio [106] Association of Aβ42/Aβ40 ratio with depression severity at 3 years (HAMD score) [122] Positive correlation between Aβ42 levels and depressive symptoms (SGDS-K score) [126] ↑ depressive symptoms at 4-year follow-up (CES-D score) associated with ↓ Aβ40 and Aβ42 levels [127] No significant association of Aβ42/40 ratio with late-life depressive symptoms (GDS-15 score) [129]
		Tau	Limited	↑ Tau/Aβ42 ratio associated with ↓ DSST score [106]	Positive association of p-tau181 levels with ↑ depressive symptoms (GDS score) [96] No significant association of p-tau181 levels with late-life depressive symptoms (GDS-15 score) [129]
Bipolar disorder (BD)	Verbal learning, memory, attention, executive function, psychomotor speed	NfL	Yes	Inverse correlation with mean time of 2-back working memory task [93] Negative association with composite score on BAC-A test, particularly verbal fluency and processing speed, in young BD patients [134] Positive association with verbal fluency and motor speed in old BD patients (BAC-A test) [134] No association with global cognition, verbal memory, executive function, psychomotor speed, sustained attention [141]	Positive association with illness duration [137]
		GFAP	Lack of evidence	/	
		Aβ	Limited	No association between Aβ42, Aβ40 levels and global cognition, verbal memory, executive function, psychomotor speed, sustained attention [141]	
		Tau	Limited	No association between total tau levels and global cognition, verbal memory, executive function, psychomotor speed, sustained attention [141]	
Schizophrenia (SZ)	Processing speed, memory, attention, executive function	NfL	Inconsistent	No significant relationship with cognitive function on RBANS test [147] Inverse correlation with global functioning (GAF scale) in acutely ill unmedicated SZ [146]	Positive correlation with symptom severity (PANSS score) in acutely ill unmedicated SZ [146] Negative association with negative symptom severity in clinical high-risk SZ [147] Positive correlation with illness duration [90]
		GFAP	Lack of evidence	/	
		Aβ	Lack of evidence	/	
		Tau	Limited	↑ p-tau levels associated with ↑ working memory performance (Digit Span test) in SZ treated with active tDCS [154]	

Abbreviations: Aβ, amyloid beta; AFT, Animal Fluency Test; BAC-A, Brief Assessment of Cognition in Affective Disorders; BD, bipolar disorder; CES-D, Centre for Epidemiological Studies—Depression scale; DSST, Digit Symbol Substitution Task; GAF, Global Assessment of Functioning; GDS, Geriatric Depression Scale; GFAP, glial fibrillary acidic protein; HADS-D, Hospital Anxiety and Depression Scale—subscale D; HAMD, Hamilton Depression Rating Scale; MADRS, Montgomery–Åsberg Depression Rating Scale; MDD, major depressive disorder; MMSE, Mini-Mental State Examination; MoCA, Montreal Cognitive Assessment; NfL, neurofilament light chain; PANSS, Positive and Negative Syndrome Scale; p-tau, phosphorylated tau; RBANS, Repeatable Battery for Assessment of Neuropsychological Status; SGDS-K, Short Geriatric Depression Scale—Korean version; SZ, schizophrenia; tDCS, transcranial Direct Current Stimulation. Arrows indicate direction of change: ↑ increase, ↓ decrease.

## Data Availability

No new data were created or analyzed in this study. Data sharing is not applicable to this article.

## Abbreviations

The following abbreviations are used in this manuscript:

Aβ	Amyloid beta
AD	Alzheimer’s disease
AUC	Area under the curve
BD	Bipolar disorder
bvFTD	Behavioral variant of frontotemporal dementia
CSF	Cerebrospinal fluid
DLB	Dementia with Lewy bodies
DSM-5-TR	Diagnostic and Statistical Manual of Mental Disorders, 5th edition, text revision
DSST	Digit Symbol Substitution Task
FTD	Frontotemporal dementia
FTLD	Frontotemporal lobar degeneration
GAF	Global Assessment of Functioning
GFAP	Glial fibrillary acidic protein
HC	Healthy controls
MCI	Mild cognitive impairment
MDD	Major depressive disorder
MMSE	Mini-Mental State Examination
MoCA	Montreal Cognitive Assessment
NfL	Neurofilament light chain
P-tau	Phosphorylated tau
PANSS	Positive and Negative Syndrome Scale
PPD	Primary psychiatric disorders
RBANS	Repeatable Battery for Assessment of Neuropsychological Status
SMIs	Severe mental illnesses
SZ	Schizophrenia
tDCS	Transcranial Direct Current Stimulation
